# Prior Puma Lentivirus Infection Modifies Early Immune Responses and Attenuates Feline Immunodeficiency Virus Infection in Cats

**DOI:** 10.3390/v10040210

**Published:** 2018-04-20

**Authors:** Wendy S. Sprague, Ryan M. Troyer, Xin Zheng, Britta A. Wood, Martha Macmillan, Scott Carver, Susan VandeWoude

**Affiliations:** 1 Department of Microbiology, Immunology and Pathology, College of Veterinary Medicine and Biological Sciences, Colorado State University, Fort Collins, CO 80523, USA; rtroyer@uwo.ca (R.M.T.); zhen0074@rams.colostate.edu (X.Z.); brittawood@gmail.com (B.A.W.); mrsmarthamac@gmail.com (M.M.); scott.carver@utas.edu.au (S.C.); Sue.Vandewoude@ColoState.EDU (S.V.); 2 Sprague Medical and Scientific Communications, LLC, Fort Collins, CO 80528, USA; 3 Department of Microbiology and Immunology, University of Western Ontario, London, ON N6A5C1, Canada; 4 The Pirbright Institute, Pirbright, Surrey GU24 0NF, UK; 5 School of Natural Sciences, University of Tasmania, Hobart, Tasmania 7001, Australia

**Keywords:** feline immunodeficiency virus (FIV), puma lentivirus (PLV), innate immunology, CD8, FAS (death receptor, CD95)

## Abstract

We previously showed that cats that were infected with non-pathogenic Puma lentivirus (PLV) and then infected with pathogenic feline immunodeficiency virus (FIV) (co-infection with the host adapted/pathogenic virus) had delayed FIV proviral and RNA viral loads in blood, with viral set-points that were lower than cats infected solely with FIV. This difference was associated with global CD4^+^ T cell preservation, greater interferon gamma (IFN-γ) mRNA expression, and no cytotoxic T lymphocyte responses in co-infected cats relative to cats with a single FIV infection. In this study, we reinforced previous observations that prior exposure to an apathogenic lentivirus infection can diminish the effects of acute infection with a second, more virulent, viral exposure. In addition, we investigated whether the viral load differences that were observed between PLV/FIV and FIV infected cats were associated with different immunocyte phenotypes and cytokines. We found that the immune landscape at the time of FIV infection influences the infection outcome. The novel findings in this study advance our knowledge about early immune correlates and documents an immune state that is associated with PLV/FIV co-infection that has positive outcomes for lentiviral diseases.

## 1. Introduction

Feline immunodeficiency virus (FIV) infection, which is a pathogenic lentivirus of domestic cats, causes fatal immune dysfunction that is characterized by progressive depletion of CD4^+^ T lymphocytes that is similar to HIV infection of humans [1,2]. More than two dozen felid species have demonstrated seroreactivity against FIV antigens, and FIV that was isolated from puma (Puma concolor) has been characterized genetically as Puma lentivirus (PLV) infection in domestic cats causes a nonpathogenic disease that results in initially high viral titers, but marked diminution early in infection [3,4,5,6].

Previously, we found that cats that were infected with non-pathogenic PLV and then infected with pathogenic FIV (co-infection with the host adapted/pathogenic virus) had delayed FIV proviral and RNA viral load detection in blood, with an overall viral set-point decrease compared to cats infected solely with FIV. This difference was associated with global CD4^+^ T cell preservation and greater interferon gamma (IFN-γ) mRNA expression, but not cytotoxic T lymphocyte responses in co-infected cats relative to cats with single FIV infection [7,8]. Co-infected cats also had accelerated anti-FIV capsid antibody development soon after FIV infection when compared to cats with single PLV or single FIV infection [9], and multivariate analysis implicates immediate anti-PLV immune responses involving CD8^+^ T cells, CD25^+^ cells, IL-4, IFN-γ, and the death receptor (FAS) as correlates of attenuated disease in co-infected cats [10]. Furthermore, co-infection results in a severe bottleneck restricting virulent FIV infection in the face of prior PLV infection, as evidenced by genomic analysis, suggesting a redistribution of viral infection and viral fitness [11,12]. Taken together, we hypothesize that the differences in available cell targets and early immune activation parameters are plausible explanations for these observations. Therefore, the current study aimed to compare lymphocyte and cytokine responses in blood, bone marrow, and lymphoid tissues of cats that were acutely infected with a pathogenic FIV isolate vs. those acutely that were infected with a nonpathogenic PLV isolate, followed by co-infection with pathogenic FIV.

## 2. Materials and Methods

### 2.1. Viral Stocks

Stocks of FIV-C36 and PLV-1695 were prepared, as described previously [7]. Briefly, stocks of FIV-C virus were propagated by co-culture of retropharyngeal cells from an FIV-C positive cat and domestic cat MYA-1 cells with a final titer of 10^7.2^ tissue culture infectious dose 50 (TCID_50_)/mL. Stocks of PLV-1695 were similarly propagated by the co-culture of PLV-infected puma PBMC with domestic cat MYA-1 cells, resulting in a titer of 10^4.7^ TCID_50_/mL.

### 2.2. Animals

Twenty-four specific-pathogen-free (SPF) cats were used from a breeding colony at Colorado State University (CSU). Animals were randomized by litter and gender and they were housed in groups of six per pen in isolation rooms of an AAALAC-international accredited animal facility. All of the procedures were approved by the CSU Institutional Animal Care and Use Committee prior to initiation (approval number: 01-246A-08, 1 March 2008).

### 2.3. Study Design

The cats were divided and housed in the following four groups (*n* = 6 per group): (1) cats receiving only PLV-1965 (PLV), (2) cats receiving PLV-1695 followed by FIV-C36 one month later (CO), (3) cats receiving only FIV-C36 (FIV), and (4) cats receiving sham inoculations of media (SHAM).

**Figure 1:**
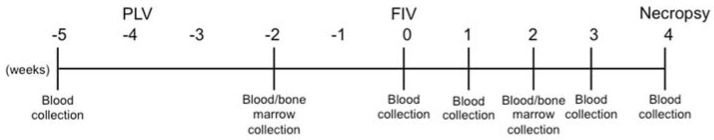
Study timeline.

Blood samples were obtained by venipuncture of the cephalic vein on conscious animals at −5, −2, 0, 1, 2, 3, and 4 weeks (post-FIV inoculation; FIV PI) (Figure 1). Bone marrow samples were collected from the humerus following ketamine/acepromazine/butorphanol anesthesia at −2 and 2 weeks FIV PI (Figure 1). At −4 weeks FIV PI, 12 26-week-old cats were inoculated intravenously (IV) with 1 mL of PLV, as previously described [7], while the remaining 12 cats received 1 mL of culture supernatant from un-infected MYA-1 cells IV. Four weeks later (week 0), six of the PLV-inoculated animals and six of the SHAM controls received 1 mL of FIV stock IV that had been diluted 1:80 in a 0.9% NaCl solution. The remaining 12 animals received 1 mL of culture supernatant from un-infected MYA-1 cells IV. The study termination was eight weeks post-PLV inoculation and four weeks post-FIV challenge. Animals were humanely euthanized, and bone marrow, thymus, and mesenteric and prescapular lymph nodes were collected at necropsy (see Figure 1 below).

### 2.4. Physical Examinations

Animals were monitored daily for clinical signs of illness, as well as general health throughout the study. Physical examinations, including weight and temperature measurements, were performed at each blood collection.

### 2.5. Cell Isolation

Cells were isolated and purified from peripheral blood, bone marrow, and tissues throughout the study for flow cytometry analysis. Peripheral blood mononuclear cells (PBMC) and bone marrow cells were purified on a Histopaque 1.077 (Sigma, St. Louis, MO, USA) gradient, according to the manufacturer’s instructions. Tissue cells were purified using a 100 µm cell strainer.

### 2.6. Hematology

Total white and red blood cell counts were measured using a Coulter Z1 (Coulter, Miami, FL, USA). One hundred-cell differential counts were performed using a microscope (Olympus BX40 clinical microscope, Center Valley, PA, USA).

### 2.7. Flow Cytometry

Percentages of PBMC and tissue cells positive for each subset examined were determined by flow cytometry using monoclonal or polyclonal antibodies (Table 1). Markers were selected to identify the significant subsets of lymphocytes, including T cells in various states of activation and maturation, and B cells (Table 2). Antibodies were conjugated to fluorochromes using Zenon kits, according to manufacturer’s instructions (Invitrogen, Carlsbad, CA, USA). 2 × 10^5^ to 1 × 10^6^ PBMCs were blocked using goat serum (MP Biomedicals, Solon, OH, USA) at a 1:10 dilution and were incubated for 30 min at 4 °C. After washing, the cells were incubated for 30 min at 4 °C with the primary antibody at varying dilutions (Table 1). Cells were then washed three times in flow buffer (phosphate buffered saline + 5% fetal bovine serum) and were resuspended in 200 µL of a buffer with 1% paraformaldehyde for fixation. Samples were analyzed on a DAKO Cyan ADP (Beckton-Dickinson, Brea, CA, USA). Gates were set to eliminate small particles, neutrophils, and eosinophils using forward and side scatter. A total of at least 10,000 cells were counted, and the percentage of cells that were stained with each antibody was determined. Gates were set based on the isotype controls (Table 1) when used at the same dilution as the antibody, such that 1% or fewer cells were positive.

List mode files were analyzed using FlowJo (Tree Star Inc., San Carlos, CA, USA). For PBMC, absolute cell counts were determined by multiplying the percent of gated cells expressing each subset by the total white blood cell count minus the absolute neutrophil, eosinophil, and basophil counts, as determined by blood smear differential counts.

### 2.8. Genomic DNA Extraction

DNA was extracted from tissues using a bead-based disruption/homogenization system and the DNeasy Blood and Tissue Kit (QIAGEN, Valencia, CA, USA). Briefly, 40 mg of tissue were placed in Lysing Matrix A tubes (M.P. Biomedicals, Irvine, CA, USA) before adding 450 μL tissue lysis buffer ATL (QIAGEN) and 50 μL proteinase K (QIAGEN). Tissues were homogenized by high-speed bead disruption in the FastPrep®-24 instrument (M.P. Biomedicals) for 40 s at a speed setting of 6.0. The resulting homogenate was centrifuged at 14,000 × *g* for 10 min, and the supernatant was transferred to a new microcentrifuge tube. DNA was extracted as per the manufacturer’s instructions. DNA was eluted with 100 μL H_2_O and stored at −20 °C until use.

DNA was extracted from 1 million PBMCs using the Qiamp blood mini DNA kit (QIAGEN). DNA from each sample was eluted with 50–100 μL of H_2_O and stored at −20 °C until use.

### 2.9. RNA Extraction & cDNA Synthesis

RNA was extracted from tissues using the FastRNA pro-green kit (M.P. Biomedicals, Irvine, CA, USA) with FastPrep^®^-24 homogenizer (M.P. Biomedicals), following the manufacturer’s protocol. Briefly, 100 mg of tissue was homogenized in RNApro™ Solution and Lysing Matrix D using the FastPrep^®^-24 instrument for 40 s at a setting of 6.0. RNA was then purified according to the manufacturer’s instructions, resuspended in 100 μL RNase-free H_2_O and stored at −80 °C. Cellular RNA was then converted to cDNA using random primers and Superscript II (Invitrogen, Carlsbad, CA, USA) according to the manufacturer’s instructions. RNA was extracted from 2 million bone marrow cells were resuspended in 0.5mL Trizol (Sigma, St. Louis, MO, USA) at 4 million cells/mL. RNA was purified by phenol:chloroform extraction and ethanol precipitation. Viral RNA was extracted from plasma using the QIAamp viral RNA kit (QIAGEN, Valencia, CA, USA) spin protocol.

### 2.10. Quantitation of Proviral Load, mRNA Viral Load, & Cytokine Transcripts by Real-Time qPCR

Briefly, 5 μL of tissue extracted DNA was quantitated by comparison to standard curves that were generated using plasmids containing the FIV-C gag or the PLV pol. The number of cell equivalents for each DNA sample was determined, as described by Terwee et al. [7]. FIV and PLV mRNA was quantified in tissues by qPCR using the previously described FIV-C gag and the PLV pol assays [13,14]. While these assays target two different lentiviral genes, the abundance of gag and pol-containing mRNAs during feline lentiviral infection are similar, suggesting that these assays are reasonable for comparing viral mRNA levels of FIV and PLV. [15] To allow for the accurate comparison between samples, viral mRNA expression was normalized to mRNA expression of glyceraldehyde-3-phosphate dehydrogenase (GAPDH) for each sample using the 2−Δ*C*t method, in which Δ*C*t is the cycle at which the threshold is reached for GAPDH that was subtracted from the cycle threshold for viral mRNA. Conversion of Δ*C*t to 2−Δ*C*t produced a value that indicates the fold abundance of viral mRNA relative to that of GAPDH mRNA.

Cytokine mRNA expression was quantitated by qPCR, as previously described [7]. Expression of IL-10, IL12p40, and IFNγ mRNA was quantitated relative to GAPDH mRNA expression using this the 2−Δ*C*t method. All of the qPCR assays in the study were performed in triplicate, and qPCR efficiencies were within the accepted range of 90–110%.

### 2.11. Statistical Analyses

To evaluate the effects of infection, time, and the interaction on the response variables, we utilized repeated measures ANOVA after the log transformation of the data. To achieve this, we split the experiment into a PLV group (at week 0), a CO group, and a SHAM group (week 0 to week 4) and analyzed these independently. Analyses were undertaken using the program R (http://www.r-project.org/). Individual time point differences between the FIV and CO groups in blood and tissues were evaluated using the Student’s *t*-test. Linear regression was used to predict the effect of previous PLV infection on decreased FIV proviral and viral loads in the CO group. *T*-tests and linear regression analyses were analyzed with PRISM software (GraphPad Software, LaJolla, CA, USA). For all of the analyses, a *P* < 0.05 was considered to be statistically significant.

## 3. Results

### 3.1. Prior PLV Infection Results in Lower FIV Proviral Loads in PBMC, Bone Marrow, and LNs, and Decreased Plasma Viremia

**Figure 2:**
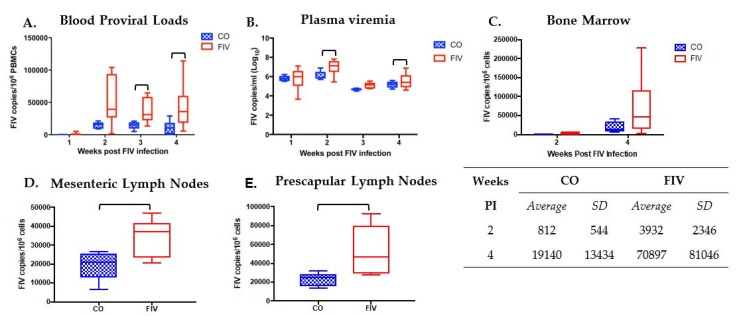
Feline immunodeficiency virus (FIV) proviral loads (**A**) and FIV plasma viremia (**B**) were statistically decreased at various times post FIV infection (FIV PI) in CO versus FIV cats. At 3 (*P* = 0.016) and 4 (*P* = 0.049) weeks FIV PI for FIV proviral loads, and at 2 (*P* = 0.048) and 4 (*P* = 0.011) weeks FIV PI for plasma viremia. However, no statistical differences were found by group and time (*P* = 0.190). Bone marrow proviral loads (**C**) were not statistically different by group and time (*P* = 0.182), but a trending difference was noted between CO and FIV cats at 2 weeks FIV PI (*P* = 0.084) and no difference was found at 4 weeks FIV PI (*P* = 0.196). Proviral loads in mesenteric (*P* = 0.0367) (**D**) and prescapular (*P* = 0.0152) (**E**) lymph nodes were lower in the CO as compared with FIV cats at 4 weeks FIV PI. Bars indicate statistical differences (*P* <0.05) between groups.

FIV proviral loads were significantly lower at three and four weeks, and FIV plasma viremia was significantly lower at two and four weeks FIV PI in CO versus FIV cats (Figure 2A,B; *p*-values < 0.05). FIV proviral loads were trending lower in the bone marrow of the CO versus FIV cats at two and four weeks FIV PI (*P* = 0.084 and *P* = 0.196, respectively; Figure 2C). Decreased proviral loads were also noted in the mesenteric and prescapular lymph nodes four weeks FIV PI in CO versus FIV cats (Figure 2D, E). Viral load differences were not found in the thymus (Supplementary Figure S1). In all of the tissues, the range of viral loads was narrower in CO than FIV cats.

### 3.2. Global and Naïve CD4^+^ T Cells are Preserved by Co-Infection Compared to FIV Infection

**Figure 3:**
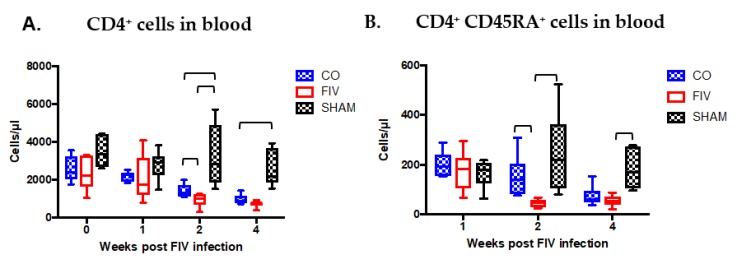
CD4^+^ cells were preserved in the blood of CO as compared with FIV cats at 2 weeks (*P* = 0.0492), and approached preservation at 4 weeks (*P* = 0.0612) FIV PI. At 2 and 4 weeks FIV PI, control SHAM cats had higher CD4^+^ cells than FIV cats (*P* = 0.002 and *P* < 0.0001, respectively), and CO cats (2 weeks, *P* = 0.0090; 4 weeks, *P* = 0.0034) (**A**). Naïve CD4^+^CD45RA^+^ cells were also preserved in the CO cats compared with FIV cats at 2 weeks (*P* = 0.0012), but not at 4 weeks (*P* = 0.332) FIV PI. At 2 and 4 weeks FIV PI, SHAM CD4^+^CD45RA^+^ cells were greater than FIV cats (2 weeks, *P* = 0.0069; 4 weeks, *P* = 0.0012), yet the SHAM was only different than the CO cats at 4 weeks FIV-PI (*P* = 0.0062) CD4^+^ cells (**B**). Bars indicate statistical differences between the indicated groups. Significant differences were noted among the three groups over time (CD4^+^ cells, *P* = 0.0004; CD4^+^CD45RA^+^ cells, *P* = 0.0012).

Global CD4^+^ cells were increased in the CO when compared to the FIV cats at 2 and 4 weeks FIV PI (*P* < 0.05, Figure 3A). CD4^+^ subset preservation was documented in the naïve CD4^+^CD45RA^+^ cell compartment at 2 weeks FIV PI in the CO verses FIV cats, but not to the same extent as the global CD4^+^ cells (Figure 3B, *P* = 0.001).CD4^+^CD45RA^+^ cells in the CO cats decreased at four weeks; and therefore, no longer differed between the groups (*P* = 0.332).

### 3.3. Prior PLV Infection Resulted in Greater CD8^+^ Cell, CD8^+^FAS^+^ cell, and large granular lymphocytes Numbers During Early FIV Infection Compared With SHAM Animals

**Figure 4:**
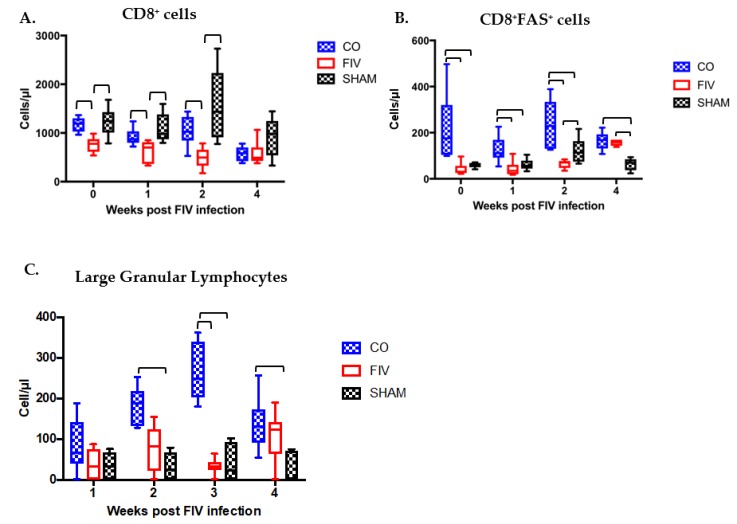
CD8^+^ cells are higher in the blood of CO compared with FIV cats at 0, 1 and 2 weeks FIV-PI (*P* = 0.0015, *P* = 0.045, *P* = 0.011, respectively). CD8^+^ cells in CO cats are similar to SHAM cats at all time points (*P* > 0.05), whereas FIV cats had decreased levels compared with SHAM cats at 0, 1 and 2 weeks FIV PI (*P* = 0.005, 0.0136, and 0.003, respectively) (**A**). CD8^+^FAS^+^ cells in the CO cats were higher than SHAM cats at all of the time points (0 week, *P* = 0.00098; 1 week, *P* = 0.0198; 2 weeks, *P* = 0.0292; 4 weeks, *P* = 0.00135), and higher in FIV as compared with SHAM cats at 2 and 4 weeks FIV PI (*P* = 0.02577, 0.00088, respectively) (**B**). Large granular lymphocyte (LGL) numbers mirrored the CD8^+^FAS^+^ cell populations, and differences were trending at one week (*P* = 0.074) and were significant at two weeks (*P* = 0.002) FIV PI between CO and FIV cats. Significant differences in LGLs were trending at one week (*P* = 0.074) and were significant at two weeks FIV PI in between the CO and FIV cats (*P* = 0.002). LGLs were higher in the CO than SHAM cats at 2, 3, and 4 FIV PI (*P* = 0.0076, 0.0059, and 0.0044, respectively). However, no significant difference was found in LGL numbers between the FIV and sham inoculations of media (SHAM) cats at any time point (**C**). Bars indicate statistical differences between the indicated groups. Significant differences were also noted among the three groups over time (CD8^+^ cells, *P* = 0.0005; CD8^+^FAS^+^ cells, *P* = 0.0004; LGLs, *P* = 0.003).

Global CD8^+^ cells (Figure 4A) were higher in the CO cats as compared with the FIV cats at 0, 1, and 2 weeks FIV PI (*p*-values <0.05); however, by 4 weeks PI all of the groups had similar CD8^+^ cell numbers. CD8^+^ cells in the CO and SHAM cats were similar at all time points (0–4 weeks), whereas the FIV cats had decreased levels compared with SHAM cats at 0, 1, and 2 weeks FIV PI (*p* < 0.05). CD8^+^FAS^+^ cell numbers were greater in the CO than FIV cats at 0, 1, and 2 weeks FIV PI (*p*-values < 0.05), and this difference was more pronounced than that seen in the global CD8^+^ cell population (Figure 4B). By four weeks FIV PI, CD8^+^FAS^+^ cells in the FIV cats increased. CD8^+^FAS^+^ cells in the CO cats were higher than the SHAM cats at all of the time points (0–4 weeks, *P* <0.05), and the FIV cats had higher levels as compared with SHAM cats at 2 and 4 weeks FIV PI (*P* <0.05). As previously noted [13], large granular lymphocyte (LGL)numbers mirrored the CD8^+^FAS^+^ cell populations, and significant differences were trending at one week (*P* = 0.074) and were significant at two weeks FIV PI between the CO and FIV cats (*P* = 0.002; Figure 4C). LGLs were higher in the CO versus SHAM cats at 2, 3 and 4 FIV PI (*P* <0.05). However, no significance was found in LGL numbers between the FIV and the SHAM cats at any time point.

### 3.4. Cytokine Differences in PBMC Between Co-Infected and FIV-Infected Cats FIV PI

**Figure 5:**
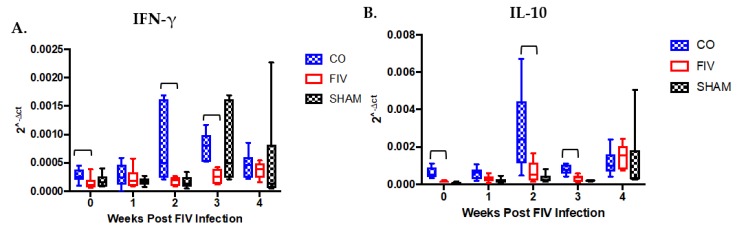
Peripheral blood mononuclear cell (PBMC) interferon gamma (IFN-γ) expression was higher in the CO as compared to FIV cats at 0, 2 and 3 weeks FIV PI (*p*-values <0.05) (**A**), while no differences were found among the groups over time (*P* = 0.2269). PBMC IL-10 mRNA expression was appreciated at 0, 2, and 3 weeks FIV PI in the CO when compared with FIV cats (*P* = 0.003), 2 (*P* = 0.044), and 3 (*P* = 0.005) Significant differences were noted for IL-10 expression among the three groups over time (*P* = 0.0001) (**B**). Bars indicate statistical differences between the CO and FIV cats.

PBMC IFN-γ expression was significantly higher in CO than FIV cats at 0, 2, and 3 weeks FIV PI (*p*-values < 0.05; Figure 5A), despite no overall differences among the groups over time. IL-10 mRNA expression was significantly different among all groups over time, and significantly higher IL-10 expression was appreciated at 0, 2, and 3 weeks (*p*-values < 0.05) in CO as compared with FIV cats (Figure 5B). No differences were seen among groups for IL-12 mRNA expression (Supplementary Figure S2).

### 3.5. Tissue Lymphocyte Phenotypes Differ Between Co-Infected and FIV-Infected Cats

#### 3.5.1. Thymus

**Figure 6:**
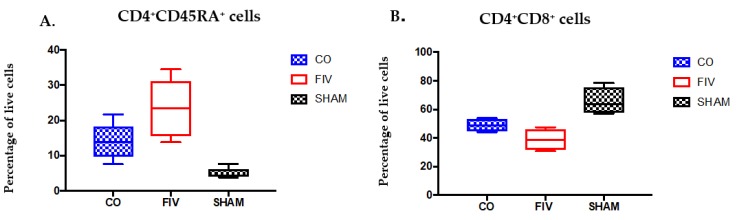
Thymic CD4^+^CD45RA^+^ cell percentages were trending lower in the CO compared to FIV cats (*P* = 0.059), and both the FIV (*P* <0.0001) and CO cats (*P* = 0.0014) had higher CD4^+^CD45RA^+^ live cell percentages than the SHAM cats at 4 weeks FIV PI (**A**). CD4^+^CD8^+^ double positive cell percentages in the CO cats were trending higher when compared with those in the FIV cats (*P* = 0.071), and both the FIV (*P* = 0.004) and CO (*P* = 0.0165) cats had lower CD4^+^CD8^+^ double positive cells compared with SHAM cats (**B**). Bars indicate statistical differences between the indicated groups.

At 4 weeks FIV PI, Thymic CD4^+^CD45RA^+^ cell percentages among live cells were trending lower in the CO when compared to FIV cats (*P* = 0.059, Figure 6A), and both the FIV and CO cats (*p*-values < 0.05) had higher CD4^+^CD45RA^+^ cell percentages than SHAM cats. CD4^+^CD8^+^ double positive cell percentages in the CO cats were also trending higher as compared with the FIV cats (*P* = 0.071, Figure 6B), and both the FIV (*P* = 0.004) and the CO (*P* = 0.0165) cats had lower CD4^+^CD8^+^ double positive cells compared with the SHAM cats. No differences were found in the thymus among single CD4^+^ and CD8^+^ cells between FIV and CO cats (Supplementary Figure S3).

#### 3.5.2. Prescapular Lymph Node

**Figure 7:**
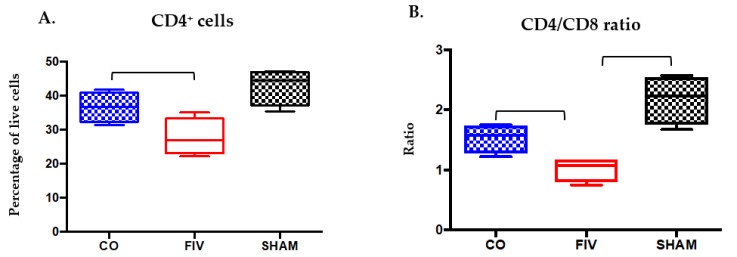
Prescapular lymph node CD4^+^ live cell percentages ((**A**) *P* = 0.0475) and CD4/CD8 ratios ((**B**) *P* = 0.02) were higher in CO when compared to FIV cats at 4 weeks FIV PI. CD4^+^ live cell percentages and CD4/CD8 ratios were also higher in the SHAM compared to FIV cats (CD4^+^: *P* = 0.008; CD4/CD8 ratio: *P* = 0.0011), but not between the SHAM and the CO cats.

At 4 weeks FIV PI, and similar to observations in the peripheral blood, CD4^+^ cell percentages were significantly preserved in prescapular lymph nodes (PLNs) in CO when compared to FIV cats (*P* = 0.0475, Figure 7A). While the CD4^+^ cell percentages in the CO cats were not different from the SHAM cats (*P* > 0.05), the FIV cats were different (*P* < 0.05). While CD8^+^ cell percentages did not vary among groups (data not shown), the CD4/CD8 ratio differences were significantly lower in the FIV when compared with CO cats (*P* = 0.020, Figure 7B), presumably due to the differences in the CD4^+^ cells between the groups.

### 3.6. PLV Infection Alters the Immune Landscape at the Time of FIV Inoculation

**Figure 8:**
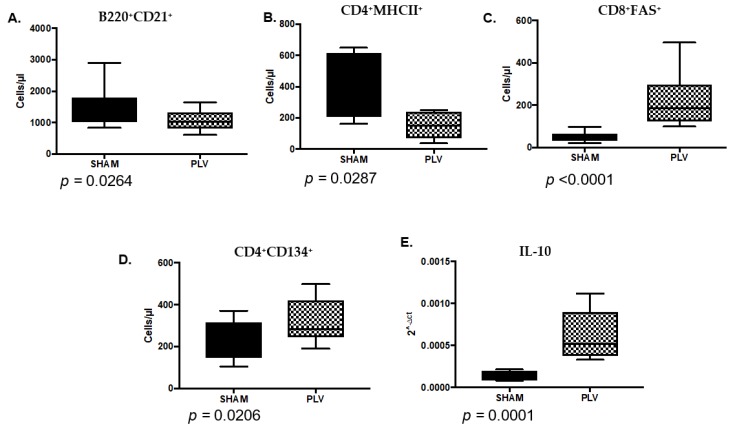
Significantly different blood cells and cytokines are seen between Puma lentivirus (PLV)-infected (PLV) and control cats (SHAM) four weeks after PLV inoculation (at week 0 FIV PI). B220^+^ CD21^+^ cells (**A**) and CD4^+^MHC II^+^ cells (**B**) were decreased in the PLV cats compared to the SHAM cats. CD8^+^FAS^+^ cells (**C**) and CD4^+^CD134^+^ cells (**D**) were increased in the PLV cats when compared to SHAM cats. PBMC IL-10 mRNA expression (**E**) was statistically increased in PLV compared to SHAM cats.

To understand the possible reasons for attenuation of FIV proviral and the viral loads in CO versus FIV cats, we evaluated blood cell and cytokine differences between the groups four weeks after PLV inoculation (at week 0 FIV PI). After four weeks of PLV infection, the cats in the PLV group had decreased B220^+^CD21^+^ B cells (Figure 8A, *P* = 0.026) and CD4^+^MHC II^+^ (Figure 8B, *P* = 0.0287) cells, and increased CD8^+^FAS^+^ (Figure 8C, *P* < 0.0001) and CD4^+^CD134^+^ cells as compared with SHAM cats (Figure 8D, *P* = 0.0206). IL-10 (Figure 8E, *P* = 0.0001) expression was also increased in PBMC at this time point.

### 3.7. Significant Differences Between Co- and FIV-Infected Cats at the Time of FIV Exposure Predict Future Outcomes of FIV Infection

**Figure 9:**
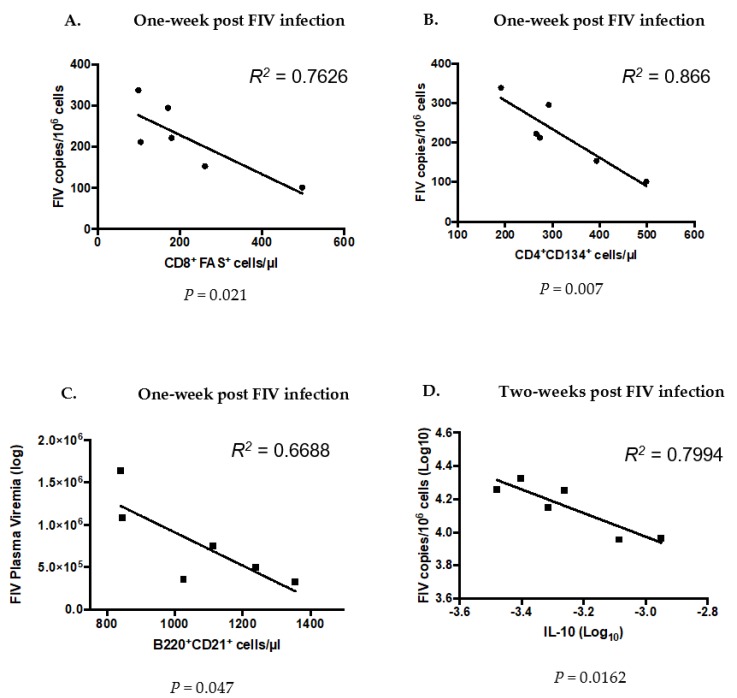
Linear regression analysis to determine cell phenotypes and cytokine expression that was different between the PLV (CO) and SHAM cats at week zero or time of FIV inoculation could predict future outcomes of FIV infection in CO cats. Higher CD8^+^FAS^+^ (**A**) and CD4^+^CD134^+^ (**B**) cell numbers in the blood of CO cats (those previously infected with PLV) at week 0 could predict lower FIV proviral loads in blood at one week FIV-PI, and that higher B220^+^CD21^+^ (**C**) cell numbers in blood at week zero could predict lower FIV plasma viremia at one week FIV PI. Higher IL-10 mRNA expression in PLV-cats at week 0 predicted lower FIV proviral loads at two weeks FIV PI (**D**).

We performed linear regression analysis to determine if the cell phenotypes and cytokines that we found to be different between the PLV cats (CO) and SHAM cats at the time of FIV inoculation (week zero) correlated with the immune or viral attributes of FIV infection. Among the cells that were different between FIV and CO cats at time zero, we found that higher CD8^+^FAS^+^ (Figure 9A) and CD4^+^CD134^+^ (Figure 9B) cell numbers in PLV-infected cats at time 0 could predict lower FIV proviral loads in blood at one week FIV PI. We also found that higher B220^+^CD21^+^ (Figure 9C) cell numbers at week zero could predict lower FIV plasma viremia at one week FIV PI and higher IL-10 expression could predict lower FIV proviral loads in blood two weeks FIV PI (Figure 9D). Cell phenotypes at week 0 were not predictive of viral loads, viremia, or circulating immunocyte populations at four weeks FIV PI.

## 4. Discussion

In previous studies, we documented that prior exposure to apathogenic PLV infection resulted in the preservation of CD4^+^ T cells and increased PBMC IFN-γ mRNA expression when compared with cats that were infected solely with FIV [7]. Increased pro-inflammatory cells and mediators [10], and attenuated proviral loads in the CO vs. FIV cats [7] were seen three weeks FIV PI. However, adaptive immune responses raised against PLV were not implicated in these differences, and G to A substitutions, due to cytidine deaminase activity, were also not found to be responsible for the bottleneck of FIV infection three weeks post-PLV infection [11,12].

The current study was designed to identify immunologic phenotype variations between CO and FIV cats during acute viral replication and to gain a better understanding of the potential mechanisms underlying the differences between these two scenarios. We again noted a blunting of FIV viral loads in blood and peripheral tissues and a dampening of CD4 depletion when PLV infection preceded FIV challenge. We hypothesized this could be due to changes in the susceptible target cell population at the time of FIV challenge in PLV versus naïve cats, and/or this impact could be due to the immunological state induced by PLV infection. While we found evidence of peripheral cell subset changes on the day of challenge, the preponderance of evidence suggests that the innate immune landscape at the time of challenge contributed to different infection outcomes.

Circulating CD4^+^CD134^+^ cells (activated CD4 T lymphocytes) and B cells varied between PLV and SHAM cats at the time of FIV challenge. CD4^+^CD134^+^ cells were higher in PLV versus SHAM cats at the time of challenge (Figure 8), a finding that correlated with lower FIV viral loads one week FIV PI (Figure 9). While CD4^+^CD134^+^ cells represent the primary cell type infected during acute FIV infection [7,16,17], the expansion of this population during PLV infection did not result in higher viral loads following FIV exposure. It is possible that this subset of activated CD4^+^ lymphocytes possessed anti-viral activity that contributed to a lower viral setpoint [18]. PLV-infected cats had statistically lower numbers of B cells in circulation on the day of FIV challenge than the SHAM cats. It is possible PLV triggered B cell apoptosis or B cell shifting from peripheral to central sites. FIV has been shown to infect CD21^+^ B cells, so lower circulating B cells in PLV could result in the decreased susceptibility to FIV challenge, though B cell tropism is typically considered to occur late in FIV infection [19].

CD4^+^CD45RA^+^ cells rapidly decreased in the FIV cats by two weeks FIV PI, but transient preservation was seen in CO cats. These findings might be explained by the fact that CD4^+^CD45RA^+^ cells are preferentially infected during acute FIV infection that is similar to acute syncytium-inducing variants of HIV infection [20,21]. None of the CD4^+^ cell subsets that were examined in this study (CD4^+^CD45RA^+^, CD4^+^CD134^+^, and CD4^+^ MHCII) could be attributed to global CD4^+^ maintenance in CO cats. Thymic CD4^+^CD45RA^+^ cells trended lower in the CO versus FIV cats. Peripheral naïve T cells were shown to be replenished by naïve thymic T cells in mice [22], and similar mechanisms may underlie our observations in this system. The lower thymic double positive cell percentages in the CO versus FIV cats might indicate a greater infection in the thymus of FIV compared with CO cats, but this did not correlate with our findings of no difference in proviral loads between the two groups. By 26 days post simian/human immunodeficiency virus, infection in rhesus macaques marked decreases in double positive cells were seen in the thymus [23]. However, differences could be due to differential changes in other thymic cell types.

CD8^+^FAS^+^ T cells were increased at the time of FIV inoculation in PLV when compared to SHAM cats, and this increase is inversely correlated with viral loads at one week PI (Figure 9A) suggesting an acute impact of these cells on the establishment of viral infection. We previously determined that CD8^+^FAS^+^ cells were analogous to LGLs in FIV and that these cells correlated with dampening of FIV viremia [24,25]. We believe that these cells are analogous to anti-viral CD8^+^ T cells in HIV-infected individuals [26] and CD8^+^ T cells in SIV-infected long-term non-progressing (LTNP) rhesus macaques [27]. Our findings bolster the conclusion that CD8^+^ T cells represent an innate immune cell type [27].

IL-10 expression at the time of FIV infection was greater in the PLV when compared to SHAM cats (Figure 9E), and the predicted suppression of proviral loads two weeks FIV PI in the CO cats. IL-10 is a complex cytokine that is induced during acute HIV infection and has potent anti-inflammatory properties in chronic HIV [28]. Higher IFN-γ expression was identified in CO when compared to FIV cats at several points post-FIV infection. Activated CD8^+^ T cells, CD4^+^ T cells, NK cells, and CD8^+^FAS^+^ cells primarily produce IFN-γ during HIV-1 and FIV infection [29,30,31,32,33]. This type II interferon has been associated with variable expression during acute and chronic HIV infection, and has not been correlated with a significant control of disease [34]. In SIV-infected sooty mangabey monkeys (SMM), IFN-γ elevation was more transient, and IL-10 was more prominent during acute control of SIV infection compared SIV infected macaques that did not control infection [35]. Although we were unable to detect differences between CD4^+^CD25^+^ between FIV and CO cats in this study, in comparison to SMM infection indicates that IL-10 expression during acute infection may be more relevant to subsequent viral control than IFN-γ. Similarly, regulatory T cell-induced IL-10 expression was shown to be responsible for decreased IFN-γ expression following the stimulation of CD4^+^ and CD8^+^ HIV-1-specific T cell immune responses of HIV-1-exposed-uninfected infants [36]. Overall, these findings show a complex interplay of cytokines that vary in intensity and at the stage of lentivirus infection, providing evidence that innate immune processes are associated with the early suppression of viral replication during co-infection.

This study reinforced previous observations that prior exposure to an apathogenic lentivirus infection can diminish the effects of acute infection with a second, more virulent, viral exposure. In addition, it investigated immunocyte phenotypes and cytokines that could distinguish the characteristics between PLV/FIV and FIV during the acute phases of the infection. Overall, circulating blood cell types at the time of infection are less relevant to FIV susceptibility than the inflammatory immune environment that is induced by prior lentiviral exposure. Additionally, as virus attenuation in HIV and SIV long-term non-progression is at least partly due to innate viral immunity [27,37], we have also identified non-virus-specific entities at play at the time of FIV inoculation in PLV-infected cats. These innate immune parameters (i.e., cytokines, LGLs/CD8^+^FAS^+^ cells) that are present during the first week of infection are key to defining differences in infection outcomes and could be used in therapeutic interventions to reduce disease severity in susceptible populations. Follow-up studies will evaluate non-infectious means to produce these biologically relevant and resistance phenotypes.

## Figures and Tables

**Table 1:** Antibodies used for flow cytometry.

Cell Receptor	Species Against	Source Antibody Clone	Isotype	Dilution
CD4	Anti-feline	Southern Biotech Clone 3-4F4	Mouse IgG1	1:100
CD8	Anti-feline	Southern Biotech Clone fCD8	Mouse IgG1	1:100
CD134	Anti-feline	Serotec Clone 7D6	Mouse IgG1	1:10
B220	Anti-mouse	Southern Biotech Clone RA3-6B2	Rat IgG2a	1:100
FAS	Anti-feline	R&D Systems Clone 431014	Mouse IgG1	1:500
CD21	Anti-human	BD Pharmingen Clone B-ly4	Mouse IgG1	1:5
CD45RA	Anti-feline	Calvin Johnson Auburn University	Mouse IgG1	1:7
MHC II (HLA-DR, DP, DQ)	Anti-human	BD Pharmingen Clone TÜ39	Mouse IgG1	1:10
CD25	Anti-feline	Wayne Thompkins North Carolina State University	Mouse IgG2a	1:20
CD14	Anti-human	Bio-Rad Clone TÜK4	Mouse IgG1	1:20

FAS: death receptor (CD95); MHC: major histocompatibility complex; HLA-DR: Human Leukocyte Antigen-antigen D Related; DP: DP chain; DQ: DQ chain.

**Table 2:** Cell subsets examined in the different tissue compartments and the proposed function of each phenotype.

Cell Subsets Examined	Tissue Compartments Examined	Proposed Function
CD4^+^	Blood, Thymus, MLN, PLN	T cells, helper of cytotoxic
CD8^+^	Blood, Thymus, MLN, PLN	Cytotoxic T cells, NKT cells
CD14^+^	Blood, Thymus, MLN, PLN	Monocytes, Macrophages, myeloid DC
CD4^+^ CD134^+^	Blood, Thymus, MLN, PLN	Activated CD4^+^ T cells
CD8^+^ FAS^+^	Blood	Activated CD8^+^ T cells
CD4^+^ MHC II^+^	Blood, Thymus, MLN, PLN	Activated CD4^+^ T cells
CD4^+^ CD45RA^+^	Blood, Thymus	Naïve CD4^+^ T cells
CD8^+^ CD45RA^+^	Blood, Thymus	Naïve CD8^+^ T cells
CD4^+^ CD8^+^	Thymus	Double-positive T cells
CD4^−^ CD8^−^	Thymus	Double-negative T cells
CD4^+^ CD25^+^	Blood	Activated or Regulatory CD4^+^ T cells
B220^+^ CD21^+^	Blood	B cells

MLN = mesenteric lymph node, PLN = peripheral lymph node.

## Supplementary Materials

Supplementary materials can be found at http://www.mdpi.com/1999-4915/10/4/210/s1.

## References

[1] Novotney C., English R.V., Housman J., Davidson M.G., Nasisse M.P., Jeng C.R., Davis W.C., Tompkins M.B. (1990). Lymphocyte population changes in cats naturally infected with feline immunodeficiency virus. AIDS.

[2] Walker C., Canfield P.J., Love D.N. (1994). Analysis of leucocytes and lymphocyte subsets for different clinical stages of naturally acquired feline immunodeficiency virus infection. Vet. Immunol. Immunopathol..

[3] Lee J.S., Bevins S.N., Serieys L.E., Vickers W., Logan K.A., Aldredge M., Boydston E.E., Lyren L.M., McBride R., Roelke-Parker M. (2014). Evolution of puma lentivirus in bobcats (*Lynx rufus*) and mountain lions (*Puma concolor*) in north america. J. Virol..

[4] VandeWoude S., Apetrei C. (2006). Going wild: Lessons from naturally occurring T-lymphotropic lentiviruses. Clin. Microbiol. Rev..

[5] Terwee J.A., Yactor J.K., Sondgeroth K.S., Vandewoude S. (2005). Puma lentivirus is controlled in domestic cats after mucosal exposure in the absence of conventional indicators of immunity. J. Virol..

[6] VandeWoude S., Hageman C.L., Hoover E.A. (2003). Domestic cats infected with lion or puma lentivirus develop anti-feline immunodeficiency virus immune responses. J. Acquir. Immune Defic. Syndr..

[7] Terwee J.A., Carlson J.K., Sprague W.S., Sondgeroth K.S., Shropshire S.B., Troyer J.L., VandeWoude S. (2008). Prevention of immunodeficiency virus induced CD4+ T-cell depletion by prior infection with a non-pathogenic virus. Virology.

[8] Zheng X., Carver S., Troyer R.M., Terwee J.A., VandeWoude S. (2011). Prior virus exposure alters the long-term landscape of viral replication during feline lentiviral infection. Viruses.

[9] Wood B.A., Carver S., Troyer R.M., Elder J.H., VandeWoude S. (2013). Domestic cat microsphere immunoassays: Detection of antibodies during feline immunodeficiency virus infection. J. Immunol. Methods.

[10] Roy S., Lavine J., Chiaromonte F., Terwee J., VandeWoude S., Bjornstad O., Poss M. (2009). Multivariate statistical analyses demonstrate unique host immune responses to single and dual lentiviral infection. PLoS ONE.

[11] Padhi A., Ross H., Terwee J., Vandewoude S., Poss M. (2010). Profound differences in virus population genetics correspond to protection from cd4 decline resulting from feline lentivirus coinfection. Viruses.

[12] Liu Y., Chiaromonte F., Ross H., Malhotra R., Elleder D., Poss M. (2015). Error correction and statistical analyses for intra-host comparisons of feline immunodeficiency virus diversity from high-throughput sequencing data. BMC Bioinform..

[13] Sondgeroth K., Leutenegger C., Vandewoude S. (2005). Development and validation of puma (*Felis concolor*) cytokine and lentivirus real-time pcr detection systems. Vet. Immunol. Immunopathol..

[14] Pedersen N.C., Leutenegger C.M., Woo J., Higgins J. (2001). Virulence differences between two field isolates of feline immunodeficiency virus (FIV-APetaluma and FIV-CPGammar) in young adult specific pathogen free cats. Vet. Immunol. Immunopathol..

[15] Ertl R., Birzele F., Hildebrandt T., Klein D. (2011). Viral transcriptome analysis of feline immunodeficiency virus infected cells using second generation sequencing technology. Vet. Immunol. Immunopathol..

[16] Shimojima M., Miyazawa T., Ikeda Y., McMonagle E.L., Haining H., Akashi H., Takeuchi Y., Hosie M.J., Willett B.J. (2004). Use of CD134 as a primary receptor by the feline immunodeficiency virus. Science.

[17] Schnittman S.M., Psallidopoulos M.C., Lane H.C., Thompson L., Baseler M., Massari F., Fox C.H., Salzman N.P., Fauci A.S. (1989). The reservoir for HIV-1 in human peripheral blood is a T cell that maintains expression of CD4. Science.

[18] Huddleston C.A., Weinberg A.D., Parker D.C. (2006). OX40 (CD134) engagement drives differentiation of CD4+ T cells to effector cells. Eur. J. Immunol..

[19] Dean G.A., Reubel G.H., Moore P.F., Pedersen N.C. (1996). Proviral burden and infection kinetics of feline immunodeficiency virus in lymphocyte subsets of blood and lymph node. J. Virol..

[20] Carreno A.D., Mergia A., Novak J., Gengozian N., Johnson C.M. (2008). Loss of naive (CD45RA+) CD4+ lymphocytes during pediatric infection with feline immunodeficiency virus. Vet. Immunol. Immunopathol..

[21] Blaak H., van’t Wout A.B., Brouwer M., Hooibrink B., Hovenkamp E., Schuitemaker H. (2000). In vivo HIV-1 infection of CD45RA(+)CD4(+) T cells is established primarily by syncytium-inducing variants and correlates with the rate of CD4(+) T cell decline. Proc. Natl. Acad. Sci. USA.

[22] Den Braber I., Mugwagwa T., Vrisekoop N., Westera L., Mogling R., de Boer A.B., Willems N., Schrijver E.H., Spierenburg G., Gaiser K. (2012). Maintenance of peripheral naive T cells is sustained by thymus output in mice but not humans. Immunity.

[23] Miyake A., Ibuki K., Enose Y., Suzuki H., Horiuchi R., Motohara M., Saito N., Nakasone T., Honda M., Watanabe T. (2006). Rapid dissemination of a pathogenic simian/human immunodeficiency virus to systemic organs and active replication in lymphoid tissues following intrarectal infection. J. Gen. Virol..

[24] Sprague W.S., TerWee J.A., VandeWoude S. (2010). Temporal association of large granular lymphocytosis, neutropenia, proviral load, and fasl mrna in cats with acute feline immunodeficiency virus infection. Vet. Immunol. Immunopathol..

[25] Sprague W.S., Apetrei C., Avery A.C., Peskind R.L., Vandewoude S. (2015). Large granular lymphocytes are universally increased in human, macaque, and feline lentiviral infection. Vet. Immunol. Immunopathol..

[26] Killian M.S., Johnson C., Teque F., Fujimura S., Levy J.A. (2011). Natural suppression of human immunodeficiency virus type 1 replication is mediated by transitional memory CD8+ T cells. J. Virol..

[27] Javed A., Leuchte N., Neumann B., Sopper S., Sauermann U. (2015). Noncytolytic CD8+ cell mediated antiviral response represents a strong element in the immune response of simian immunodeficiency virus-infected long-term non-progressing rhesus macaques. PLoS ONE.

[28] Blackburn S.D., Wherry E.J. (2007). Il-10, T cell exhaustion and viral persistence. Trends Microbiol..

[29] Fan J., Bass H.Z., Fahey J.L. (1993). Elevated IFN-gamma and decreased IL-2 gene expression are associated with HIV infection. J. Immunol..

[30] Zimmerli S.C., Harari A., Cellerai C., Vallelian F., Bart P.A., Pantaleo G. (2005). HIV-1-specific IFN-gamma/IL-2-secreting CD8 T cells support CD4-independent proliferation of HIV-1-specific CD8 T cells. Proc. Natl. Acad. Sci. USA.

[31] Feng Y.M., Wan Y.M., Liu L.X., Qiu C., Ma P.F., Peng H., Ruan Y.H., Han L.F., Hong K.X., Xing H. (2010). HIV-specific IL-2(+) and/or IFN-gamma(+) CD8(+) T cell responses during chronic HIV-1 infection in former blood donors. Biomed. Environ. Sci. BES.

[32] Montoya C.J., Velilla P.A., Chougnet C., Landay A.L., Rugeles M.T. (2006). Increased IFN-gamma production by NK and CD3+/CD56+ cells in sexually HIV-1-exposed but uninfected individuals. Clin. Immunol..

[33] Dean G.A., Pedersen N.C. (1998). Cytokine response in multiple lymphoid tissues during the primary phase of feline immunodeficiency virus infection. J. Virol..

[34] Roff S.R., Noon-Song E.N., Yamamoto J.K. (2014). The significance of interferon-gamma in HIV-1 pathogenesis, therapy, and prophylaxis. Front. Immunol..

[35] Kornfeld C., Ploquin M.J., Pandrea I., Faye A., Onanga R., Apetrei C., Poaty-Mavoungou V., Rouquet P., Estaquier J., Mortara L. (2005). Antiinflammatory profiles during primary siv infection in african green monkeys are associated with protection against aids. J. Clin. Investig..

[36] Legrand F.A., Nixon D.F., Loo C.P., Ono E., Chapman J.M., Miyamoto M., Diaz R.S., Santos A.M., Succi R.C., Abadi J. (2006). Strong HIV-1-specific T cell responses in HIV-1-exposed uninfected infants and neonates revealed after regulatory T cell removal. PLoS ONE.

[37] Killian M.S., Teque F., Walker R.L., Meltzer P.S., Killian J.K. (2013). CD8(+) lymphocytes suppress human immunodeficiency virus 1 replication by secreting type I interferons. J. Interferon Cytokine Res..

