# Supplementary material for: Prior Puma Lentivirus Infection Modifies Early Immune Responses and Attenuates Feline Immunodeficiency Virus Infection in Cats
Source: Viruses. 2018 Apr 20;10(4):210. doi: 10.3390/v10040210 (PMC5923504; doi:10.3390/v10040210)
Supplement: Supplementary file 1 [file viruses-10-00210-s001.zip › Supplementary figure 3.docx]

Supplementary figure 3. Thymic CD4^+^ and CD8^+^ live cell percentages comparing cells in CO, FIV, and SHAM group cats.

**CD4^+^ cells**

*P* = 0.425

Percentage of live cells

**CD8^+^ cells**

*P* = 0.532

Percentage of live cells
