# Supplementary material for: Prior Puma Lentivirus Infection Modifies Early Immune Responses and Attenuates Feline Immunodeficiency Virus Infection in Cats
Source: Viruses. 2018 Apr 20;10(4):210. doi: 10.3390/v10040210 (PMC5923504; doi:10.3390/v10040210)
Supplement: Supplementary file 1 [file viruses-10-00210-s001.zip › Supplementary figure 1.docx]

**Thymic FIV proviral loads**


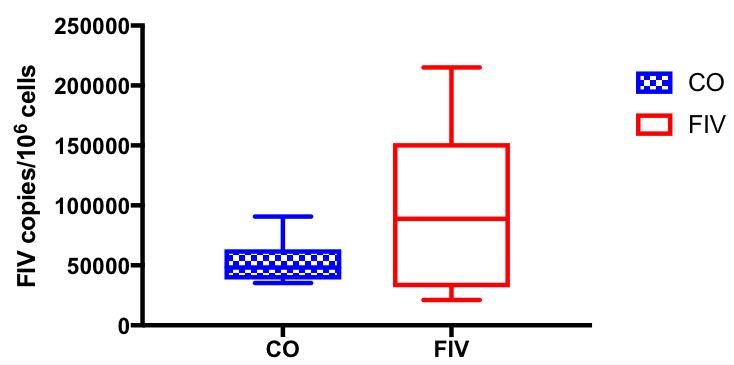


*P* = 0.4049

Supplementary figure 1. Thymic proviral loads 4 weeks post FIV infection. No differences were seen between the CO and FIV group cats.
