# Supplementary material for: Prior Puma Lentivirus Infection Modifies Early Immune Responses and Attenuates Feline Immunodeficiency Virus Infection in Cats
Source: Viruses. 2018 Apr 20;10(4):210. doi: 10.3390/v10040210 (PMC5923504; doi:10.3390/v10040210)
Supplement: Supplementary file 1 [file viruses-10-00210-s001.zip › Supplementary figure 2.docx]

**PBMC IL-12 mRNA**

*P = 0.4065*

Supplementary figure 2. PBMC IL-12 mRNA expression during weeks 0, 1, 2, 3 and 4 weeks post FIV infection. No statistical differences were seen among any of the groups over time.
